# The Criminal Law Amendment Bill

**Published:** 1917-03-10

**Authors:** 


### THE CRIMINAL LAW AMENDMENT BILL.

As was said in a previous article, this Bill is a plurimis Bill. It deals with many different subjects, all of them, however, connected directly or more or less remotely with sexual irregularities. On the publication of the Bill a summary of it appeared in the *Times*, and was followed by a detailed examination of its provisions by Dr. Helen Wilson. Dr. Helen Wilson is one of a number of women who issued a short time ago a kind of proclamation, *urbi et orbi*, on the subject of venereal diseases. Amongst others, it contained the following paragraphs:—

"Suggestions are sometimes made for dealing with this scourge by restrictions imposed only on women. Such efforts always have been and always must be futile.—We urge the Government and Parliament, and all persons interested in the subject to apply the following tests to every proposal dealing with this great problem:—First, will it tend to strengthen or to weaken that sense of individual responsibility in relation to sexual conduct which is the strongest and most effectual bulwark against the spread of disease?—And, secondly, can it, and will it, in actual practice be applied with impartial justice to both sexes and to all classes?—Only by measures which can satisfactorily meet these tests will the eradication of venereal diseases be brought about."

It is added that Dr. Helen Wilson, 19 Tothill Street, S.W., is prepared to receive correspondence on the subject. We have pleasure in responding to the invitation.

If not altogether clear, this pronouncement is at any rate emphatic, especially the final paragraph, and therefore we look with no small interest to Dr. Helen Wilson's criticism in the *Times*, to see how the Bill, in her opinion, stands the tests that settle whether the measure will or will not eradicate venereal diseases.

The first clause in the Bill renders a male person, and a male person only, punishable for an offence (an act of indecency with a girl under sixteen years of age) in which a female person is equally concerned, of which she may be equally guilty, and which she may have provoked and solicited. This does not appear to be an application of the measure in actual practice with impartial justice to both sexes, and therefore we look in Dr. Helen Wilson's remarks on the Bill for a scathing exposure of its partiality and injustice. We do not find any exposure, however. The gross and glaring injustice is passed over without a word of comment of any kind. Dr. Wilson assures us that it is *only* by measures that meet her tests that venereal diseases can be eradicated. Here is a measure that does not pass the test, that in fact violates it in the most flagrant manner; but Dr. Wilson does not protest against it. On the whole she approves of it.

Again, she and her colleagues say "Suggestions are sometimes made for dealing with this scourge by restrictions imposed only on women. Such efforts always have been and always must be futile." This may be true; but in this Bill suggestions are made for dealing with this scourge by restrictions imposed only on men. Does Dr. Helen Wilson hold that such restrictions always have been and always must be futile? If so, why does she not protest, in the comments on the Bill, against this provision. If not, why not? Is it because men and women do not stand upon an equal footing with respect to venereal diseases? Then how can the proposals be applied with impartial justice to both?

There is another curious feature in Dr. Helen Wilson's account of the Bill. She says the word used throughout is "person," and that therefore the provisions of the Bill would presumably apply to men as well as to women. "Nevertheless," she says, "it is difficult to avoid the conclusion that it is specially aimed at prostitutes." It is difficult, of course, to avoid this conclusion if we come to the Bill with the fixed determination to find in it, by hook or by crook, some injustice to women; but it is not so difficult to avoid this conclusion if we take the trouble to notice that the second word in the first clause in the Bill is "male," and that this clause, as well as another clause in the Bill, imposes restrictions on male persons and on male persons only.

Dr. Helen Wilson is a qualified medical practitioner, and therefore must have had some training in science, and must be capable in ordinary matters, in which her feelings are not deeply engaged, of seeing what is immediately under her nose, of reading documents without omitting their most important words, and of forming conclusions that are not flatly contradicted by the evidence; but it is manifest that in this matter she is blind to the obvious, and incapable of forming a trustworthy judgment. Women are very apt to accuse men of bias and injustice in their treatment of sexual matters, and to complain of the injustice with which women are treated; but with the example of Dr. Helen Wilson before our eyes it seems doubtful whether, if the management of these matters were left entirely to women, impartial justice would be secured.

